# Trust As the Foundation for Informed Decision‐Making in Prenatal Genetic Screening and Diagnostic Testing: A Qualitative Study

**DOI:** 10.1111/hex.70832

**Published:** 2026-08-23

**Authors:** Miller Finkelstein, Christina Collart, Lenicia Jenkins, Richard Frankel, Norah Crossnohare, John Bridges, Monique Katsuki, Susannah Rose, Marsha Michie, Megan Allyse, Michael Rothberg, Marissa Coleridge, Brownsyne Tucker‐Edmonds, Ruth M. Farrell

**Affiliations:** ^1^ Women's and Childrens' Institute, Cleveland Clinic Cleveland Ohio USA; ^2^ Indiana University, Indianapolis Indianapolis Indiana USA; ^3^ Division of General Internal Medicine, Department of Internal Medicine The Ohio State University Wexner Medical Center Columbus Ohio USA; ^4^ Division of Biostatistics and Population Health, Department of Biomedical Informatics The Ohio State University Wexner Medical Center Columbus Ohio USA; ^5^ Department of Biomedical Informatics & Center for Biomedical Ethics and Society Vanderbilt University Medical Center Nashville Tennessee USA; ^6^ Department of Bioethics Case Western Reserve University School of Medicine Cleveland Ohio USA; ^7^ Center for Value‐Based Care Research, Cleveland Clinic Cleveland Ohio USA; ^8^ Center for Personalised Genetic Healthcare, Cleveland Clinic Cleveland Ohio USA

## Abstract

**Background:**

Prenatal genetic screening and diagnostic testing (PS&D) requires pregnant patients to make time‐sensitive, high‐stakes decisions that often involve multifaceted risk assessments and deeply personal values. These decisions are becoming increasingly complex as the clinical integration of new genomic science and technologies advances alongside parallel changes in maternal‐foetal medicine, neonatology, and reproductive health services. Despite these changes, there is limited guidance on how best to prepare patients to formulate PS&D decisions with significant implications for obstetric outcomes. The goal of this study was to characterise pregnant patients' perceptions of the decision‐making process and identify facilitators and barriers for them to make informed decisions about their PS&D options.

**Methods:**

We conducted a qualitative study of pregnant and postpartum individuals receiving obstetric care within a large healthcare system. Participants, enroled across early pregnancy, late pregnancy, and postpartum periods, completed in‐depth semi‐structured interviews exploring their experiences, preferences, and needs related to PS&D decision‐making. Interviews were recorded, transcribed verbatim, and analysed by a multidisciplinary team using inductive thematic analysis, supported by iterative coding, memoing, and theme identification.

**Results:**

Most participants were < 35 years of age (48 of 61) and had a prior pregnancy (42 of 61). Four major subthemes emerged around a central concept of trust affecting medical decision‐making: (1) Trust as a means of navigating the complexity of PS&D: Patients relied heavily on their obstetric (OB) providers to interpret complex information and guide decisions, particularly as testing decisions became more consequential and time sensitive. (2) Trust as a prerequisite for informed decision‐making: Patients emphasised that psychological safety and relational continuity were necessary before they could ask questions, disclose concerns, or integrate medical information with personal values. (3) Erosion of trust due to perceived bias, judgement, or assumptions: Provider bias, whether expressed through communication style, assumptions about patient preferences, or perceived judgement, undermined trust and impeded informed, values‐aligned decisions. (4) Structural barriers in prenatal care models: Limited continuity, time constraints, and triaged group‐based care created obstacles to building trust and compounded difficulties in discussing sensitive topics across multiple providers.

**Conclusions:**

Our findings suggest that trust is a core component of informed PGT decision‐making and central to supporting patient‐centred communication amid uncertainty, cascading decisions, and evolving genomic technologies. Strategies to build and maintain trust, particularly within multi‐provider prenatal care models, are essential to enabling informed, values‐concordant prenatal decision‐making. System‐level interventions that promote continuity, unbiased communication, and structured agenda‐setting help strengthen prenatal care delivery and improve patient experience and outcomes.

**Patient or Public Contribution:**

Patients were involved in three main aspects of the study: (1) identifying the need for research that supports PS&D decision‐making through our prior work with patients as part study participation in focus groups, interviews, and survey studies, (2) contributing to the development of the interview guide through direct feedback to the instrument's questions and methods of data collection, and (3) participating in the study interview and providing their perspectives on how to support them and other pregnant patients as they face a myriad of pre and posttest decisions.

## 1 Introduction

The patient‐ provider relationship is foundational for effective healthcare delivery, setting the stage for a shared decision‐making process that enables patients to make informed, values‐reflective decisions about their medical care needs [1]. Positive patient experiences, high‐quality patient‐provider relationships, and clear communication are associated with improved patient satisfaction, stronger alignment with recommended care, and more accurate and timely diagnoses. These factors are associated not just with better obstetric outcomes but also decreased decisional regret [2, 3, 4].

Strong patient‐provider relationships are especially essential in prenatal care, where decisions are complex, time‐sensitive, and consequential for health outcomes. Decisions about prenatal genetic screening and diagnostic tests (collectively referred to as prenatal genetic tests, or PS&D) exemplify this challenge. PS&D provides information about a range of foetal genetic conditions and abnormalities, with the potential to lead to a cascade of complicated and highly personal decisions about prenatal care with potentially significant implications for a pregnant patient, the pregnancy, and the foetus [3, 4]. Conversations regarding these decisions should focus on how to personalise the information PS&D generates and must also consider the options if a serious foetal anomaly is identified [5, 6, 7, 8, 9]. All PS&D modalities require an individual assessment of risk and, in many cases, involve decisions, continued evaluations, or procedures if a serious foetal abnormality is identified. Such decisions require patients to balance personal beliefs and values, understand risks and probabilities, determine the potential utility of the information these tests can provide, consider care pathways, and evaluate their tolerance for uncertainty [10, 11].

Given this complexity, the patient–provider relationship must support patient‐centred care that enables informed PS&D decisions. While shared decision‐making is often presented as a way to achieve that, there is little practical guidance about how to achieve that level of healthcare communication and decision‐making in a prenatal care setting. This is also a setting where obstetric providers, who see predominantly prenatal patients, have limited time, resources, and training to fully discuss PS&D options with patients [12, 13, 14]. In some current models of prenatal care delivery (e.g., Group Prenatal Care, Early Care Model), pregnant patients will see multiple providers during their antenatal course, thus learning about their options from different sources [15].

These challenges are further heightened by the rapid emergence of new genomic technologies and evolving antenatal and postnatal options when a serious foetal anomaly is detected, coupled with parallel changes in reproductive healthcare access [16, 17, 18, 19]. To address these gaps, we conducted a study to characterise pregnant patients' needs as they navigate PS&D decisions, focusing on identifying barriers, facilitators, and resources that support informed decision‐making.

## 2 Methods

We conducted a qualitative study using a combination of descriptive and interpretative approaches consistent with the Consolidated Criteria for Reporting Qualitative Research (COREQ) [20]. We utilised this hybrid approach to characterise patients' decision‐making in the unique context of PGT decisions and then gain deeper insight into how patients define and experience barriers and facilitators to making choices that meet their needs while also being evidence‐based.

### 2.1 Recruitment

We used a consecutive, purposeful sampling strategy to capture the experiences and preferences of pregnant patients at different stages of pregnancy, reflecting different stages of exposure to PS&D options and decision‐making. Early Prenatal Group: individuals with a confirmed, viable pregnancy, EGA 8‐17 weeks 6 days EGA who were recently introduced and considering prenatal genetic testing options (Group 1); Mid‐Prenatal Group: individuals who were 18 weeks gestation to delivery and undergone the 2nd trimester anatomical ultrasound or other prenatal genetic tests (Group2), and Late Prenatal‐Postpartum Group: individuals who had completed the pregnancy, 1‐12 months postpartum (Group 3). Inclusion criteria included individuals 18 years of age or older with an identified viable intrauterine pregnancy, English‐speaking, could provide consent for research participation, and had received outpatient obstetric care at the Cleveland Clinic, which serves a range of urban and rural patient populations. Study sites include a range of clinical practice settings (e.g., hospital‐based outpatient clinic, regional community clinic) staffed by obstetricians and nurse‐midwives. These sites are within a single healthcare system with standardised approaches to prenatal care and genetic testing.

**Figure 1:**
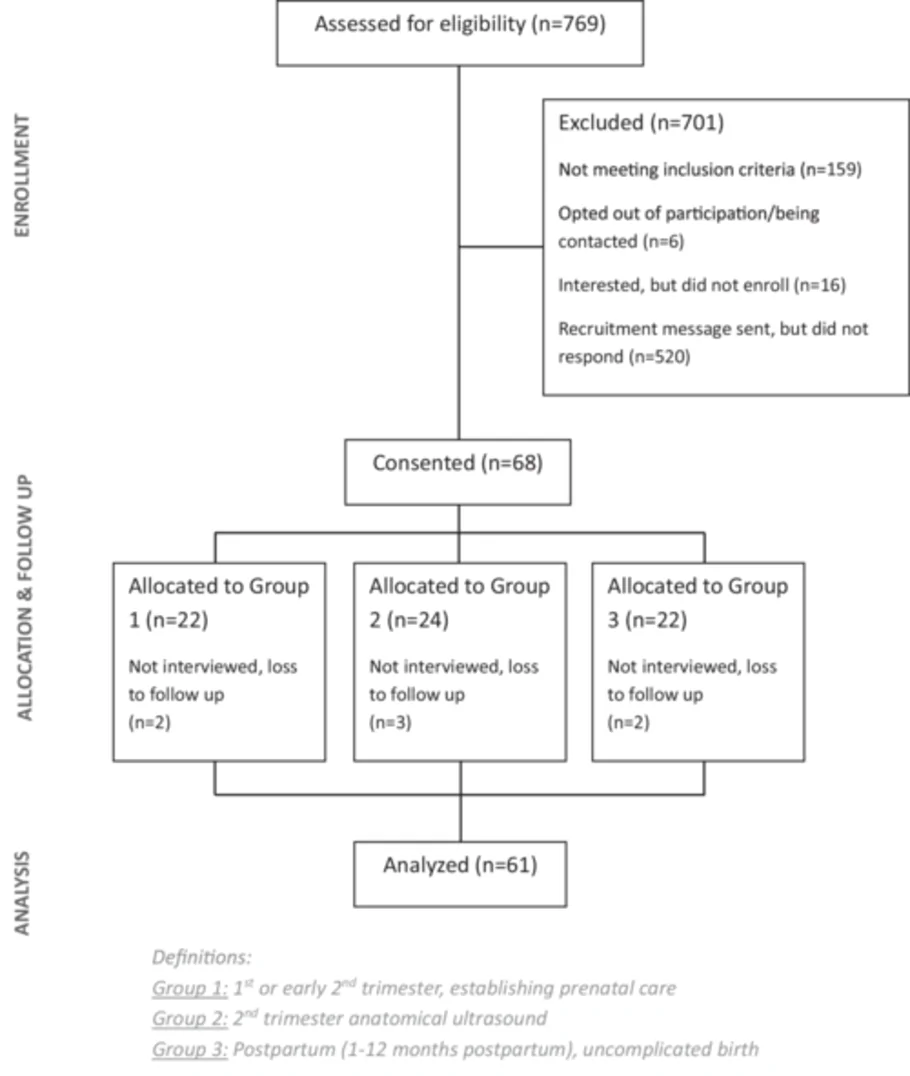
Study participant flow diagram.

Eligible participants were contacted via an IRB‐approved recruitment letter sent via a secure, electronic health record (EHR)‐based patient communication portal. The letter invited interested, eligible patients to contact the research team. Participants were enroled between January and July 2025. No participants were excluded after initial contact or enrolment; all exclusions occurred during eligibility assessment (Figure 1).

Recruitment was continued until thematic saturation was reached across the three groups. Thematic saturation was identified when new interviews across the full sample no longer added substantively new thematic content, while subgroup saturation was reached when additional interviews within each gestational age group no longer revealed new themes or important variations in experience.

### 2.2 Data Collection

During the interview process, we recognised the unique and potentially sensitive topics of pregnancy, PS&D, and parenthood, and constructed and piloted the interview guide with this issue in mind. Interviews were conducted using an interview guide developed in collaboration with content experts in obstetrics, clinical genetics, medical decision‐making, bioethics, and qualitative methods to explore PS&D decisions among pregnant and postpartum populations. The guide was pilot tested with content experts and a representative sample of participants to address issues of language, concepts, or bias that could arise in the interview process. The first part of the semi‐structured interview guide invited participants to discuss their opinions and experiences regarding the utility, benefits, and drawbacks of PS&D, their informational and decision‐making preferences, and their needs in response to a positive screen or diagnostic test result. The second part asked participants to draw on their opinions and experiences to propose recommendations for healthcare providers and other pregnant patients to better support PS&D decisions, considering technological changes and reproductive healthcare access. Interviews were recorded with corresponding field notes, transcribed verbatim, and checked for accuracy. Quantitative data were collected from the EHR regarding reproductive history, prenatal genetic test utilisation, and results at the time of the interview.

Interviews were conducted by a member of the research team (P.A.), a former genetic counsellor and doctoral‐trained researcher with expertise in women's health, genetics, and clinical research, who has extensive experience with research with pregnant patients and in discussions regarding PS&D. She used a person‐centred and nondirective approach to learning about reproductive and healthcare decision‐making to foster discussion around topics of PS&D. Participants initially met the interviewer via email to schedule the interview who introduced herself as part of the research team and not associated with healthcare providers. The interview was conducted by and via video conferencing software (Microsoft Teams) or by telephone as determined by participant preference. Field notes were collected during the interview. A consistent approach was taken with all interviews, beginning with an introductory discussion about concepts and participants' preferred language for use during the interview (e.g., the language of foetus, baby, or child), coverage across topics and question, and a closing statement to reflect on the discussion and opportunity for the participant to provide additional comments. While participants were permitted to have others present for the 45–60‐min duration of the interview, the interviewer was the only research staff member present during data collection. Participants were invited to turn off the video if they felt more comfortable discussing private and personal aspects of prenatal care off camera. The interviewer took memos during and after the interview to document their reactions to the discussion, the participants' reactions to probes and follow up questions, and emergent concepts. This approach was designed to support an in‐depth semi‐structured interview process that prioritised participants' privacy and autonomy in discussing pregnancy and PS&D. Study participation ended after the interview.

Interviews were audio recorded and professionally transcribed verbatim by a HIPAA compliant method. Transcripts were reviewed for accuracy by the research team to identify inconsistencies in how the interviewer delivered the probes or responded to participants' answers or follow‐up questions.

### 2.3 Analysis

We used thematic analysis as the primary analytic approach to identify patterns of meaning across the interview data. Transcripts were reviewed iteratively and coded to capture salient concepts related to participants' experiences and perspectives [21]. This approach was selected to allow themes to emerge from participants' narratives, particularly around unique aspects of prenatal care and PS&D decisions, while supporting systematic comparison across interviews and progressive refinement of codes and interpretations over the course of analysis. Analysis was led by two members of the research team (M.F. and R.F.) with experience in qualitative health research and interview‐based analysis. The broader multidisciplinary study team included expertise in medical decision‐making, maternal‐foetal medicine, prenatal genetics, ethics, and qualitative research, which informed interpretation of the data.

The research team met weekly throughout the analytic process to review coded transcripts, compare coding decisions, discuss analytic memos, and refine the developing thematic structure. Transcripts and field notes were reviewed repeatedly to promote immersion in the data and to identify preliminary content domains, recurrent concepts, and patterns in participants' accounts. These observations informed development of a preliminary codebook consisting of code labels, definitions, and representative examples. The codebook was refined iteratively as additional transcripts were coded, and as new concepts, distinctions, and relationships emerged from the data. Coding was conducted by M.F and R.F. using NVivo version 15. Differences in coding and interpretation were resolved through discussion and consensus. Memoing was used throughout to document analytic decisions, emerging interpretations, relationships among codes, and questions for further exploration.

Codes were then compared across transcripts and grouped into broader themes and subthemes that reflected recurring patterns in how participants described trust, communication, and decision‐making. Constant comparison across interviews was used to examine similarities and differences in participants' experiences and to clarify the boundaries, meaning, and relationships of developing themes. After themes and subthemes were identified, we drew on interpretive principles to further examine how these subthemes related to one another. This secondary analytic step was used to better understand the ways in which different dimensions of participants' experiences intersected in shaping effective healthcare communication and decision‐making. In this way, thematic analysis served as the primary framework for identifying and organising patterns in the data, while grounded theory‐informed interpretive strategies supported a more relational understanding of how those patterns fit together.

During initial coding, a primary theme of trust emerged as a recurring and cross‐cutting concept. As analysis progressed, the team recognised that trust functioned as an overarching theme in participants' descriptions of prenatal genetic testing decision‐making. To further examine this finding, we conducted a focused secondary review of full transcripts and all excerpts coded to trust to explore how trust was described, experienced, and enacted in participants' narratives. This secondary analysis demonstrated that trust was not a single static construct, but a complex phenomenon with several distinct but related dimensions. Through iterative comparison across transcripts, memo review, and team discussion, we identified four subthemes that captured these nuanced aspects of trust. Themes and subthemes were refined until consensus was reached on the final thematic structure. Throughout analysis, the team also engaged in reflexive discussion about how disciplinary perspectives, prior assumptions, and clinical or research experience might shape interpretation of the data, and these discussions informed refinement of codes and themes. Participant characteristics, including trimester of pregnancy, gravidity/parity, prenatal genetic testing use, and test results available at the time of interview, were used to contextualise interpretation of the qualitative findings. Final themes were interpreted in relation to existing literature and conceptual frameworks in medical decision‐making, with particular attention to the complexity of prenatal genetic testing decisions, which may involve a cascading set of options shaped by test results, gestational age, and patients' medical, personal, and social experiences during pregnancy.

### 2.4 Ethical Considerations

All research procedures were approved by the Cleveland Clinic IRB. Specific human subject protections were utilised to reflect the nature of research discussions regarding PGT and access to associated healthcare options. The IRB approved written informed consent from participants via DocuSign, a Part 11 Compliant electronic signature platform.

## 3 Results

A total of 61 participants were enroled, with a recruitment rate of 14.5%. Thematic saturation was achieved across the three recruitment groups: Group 1 (*n* = 20), Group 2 (*n* = 21), and Group 3 (*n* = 20) (Table 1). The mean age of participants was 32.1 years, with most participants aged < 35 years (*n* = 48) compared with those aged ≥ 35 years (*n* = 13). A greater number of participants reported their race as white (*n* = 49) and ethnicity as non‐Hispanic (*n* = 60). The majority were multigravid (G2 + ) (*n* = 42); fewer patients were primigravid (G1) (*n* = 19). Most participants had undergone either a prenatal genetic serum screening test, an ultrasound, or a diagnostic test by the time of the interview (*n* = 59), with the majority receiving a normal or average‐risk result (*n* = 57).

**Table 1:** Participant characteristics.

Factor	Total (*N* = 61)
Age	32.1 ± 4.4
Age group	
< 35	47 (78.7)
>=35	13 (21.3)
Race	
White	49 (80.3)
Black	5 (8.2)
Asian	1 (1.7)
Multiracial	3 (4.9)
Declined to answer	3 (4.9)
Ethnicity	
Hispanic or Latina	1 (1.6)
Non‐Hispanic or Latina	60 (98.4)
Gravidity	
G1	19 (31.1)
G2 or greater	42 (68.9)
Group of participants by available genetic screening/testing options	
Group 1	20 (32.8)
Group 2	21 (34.4)
Group 3	20 (32.8)

Trust emerged as the central organising theme across interviews. However, participants did not describe trust as a single, static construct; rather, their accounts reflected multiple, related dimensions of trust. Focused analysis of trust‐related narratives identified four subthemes that together characterise how trust shaped participants' interpretation of information, interactions with clinicians, and decision‐making about prenatal genetic testing: (1) trust as a component in navigating the unique complexity of PS&D decisions, (2) trust as a critical component for informed decision making, (3) the perception of provider bias, opinion, and judgement eroding trust and acting as a barrier to informed decision‐making, and (4) building and maintaining trust across different models of prenatal care delivery. Although the interview guide did not explicitly ask about trust, the concept surfaced repeatedly as participants described how they make medical decisions and where they seek information. Participants linked their ability to ask questions, interpret recommendations, and discuss sensitive preferences to whether they felt respected, heard, and not judged by their clinicians. Trust thus emerged organically as the key mechanism shaping how they evaluated information sources and engaged in prenatal genetic testing decisions. In accordance with the Standards for Reporting Qualitative Research [22], illustrative verbatim quotes are presented below, with additional data in Table 2.

**Table 2:** Additional qualitative data.

Theme	Illustrative quote
Trust as a component to navigate the unique complexity of prenatal genetic testing decisions	“I probably just rely on the doctor. Honestly, if it's too complicated for the normal person to understand, I probably would just trust the doctor's recommendation.” (G1‐22)
	“I would definitely listen to my provider and that's the best option for me because I wouldn't want to listen to anyone else.” (G2‐01)
	“I take everything on social media with a grain of salt. With having family members in the medical field, I've been taught, if I'm going to take advice from somebody it, should be somebody that has dedicated their life to studying this, is far more educated and invested in it than Instagram.” (G3‐18)
Trust as a critical component of informed decision‐making	“I know there's just some people who just aren't trusting of people, and especially medical professionals. […] I feel like they would feel like they're prejudged when they walk into that room.” (G1‐17)
	“As a mom was not being listened to, and I learned really quickly that, unfortunately, not everybody in the medical profession is on your side. Not everybody's going to hear you out. So, you have to advocate for yourself.” (G1‐17)
	“You know, it could be a provider they've been seeing since they were a teenager, and maybe they don't want to talk to them about termination and maybe they don't know how that provider feels about it.” (G2‐07)
Erosion of trust eroded by perceived provider bias, opinions, and judgement	“Even though their job as a doctor, I am sure, is to stay pretty neutral no matter what. But I guess like for her, she [her provider] read my situation. You know, I'm excited to be pregnant. Maybe she knows I don't get anxiety easily. I don't get stressed out about those things. I don't know. Maybe she just knew that I didn't need to talk about that as an option. Maybe she just used her judgement on me as a person and that went into her decision into giving me information or not, or maybe personal information, I guess I'm saying, wade in her decisions to give me information or not.” (G1‐08)
	“I kind of would worry about the judgement a little bit if I didn't know my provider as well as I knew mine personally.” (G3‐12)
	“So, it's just I think having that conversation and letting them know that they're not being judged if they decide to do certain a certain way.” (G1‐03)
Building and maintaining trust across different models of prenatal care delivery	“When I was pregnant, we had a good relationship with most of the doctors there. We were lucky enough to see most of the team at different times, and so we were able to build those relationships.” (G3‐21)
	It really is hard because you don't have a lot of time. So, you really don't get to know these people [providers], [they] don't get to know you, and you don't get to know the people to the level that I think I personally would prefer.” (G1−07)
	“I personally felt like that at some of my appointments I have not had with my preferred, my primary doctor, but I've seen other doctors when there wasn't availability for her. So that's when I felt like I was rushed or I didn't feel comfortable enough to really ask questions because I felt like it was being rushed.” (G3‐14)
	“I think it's a little bit harder to trust until you form that relationship and that comfort level with somebody.” (G1‐03)

### 3.1 Trust Is a Component in Navigating the Unique Complexity of Prenatal Genetic Testing Decisions

Participants discussed health care decisions during pregnancy as fundamentally different and more nuanced than those made outside of pregnancy and prenatal care [23]. In part, this difference was attributed to the potentially complex, *“high‐risk”* nature of the decisions. *“I think [decisions during pregnancy] definitely adds an added layer of complexity, knowing that something that might be beneficial for me might be harmful for the baby”* (G1‐11). Such decisions were described as “*high stakes*” because of the potential consequences, including significant guilt that could arise if a decision for or against testing resulted in a negative outcome. *“I think there would be some guilt associated with doing something that had so many unknowns, that if something were to happen to the foetus, and I was the one who voluntarily did this. I don't know…There would just be some sort of guilt that I would have. But, if it's just myself, that's all on me”* (G3‐20).

Participants expressed high levels of trust in clinicians to guide them through complex, high‐stakes medical decisions. Given the intricacy of medical decision‐making during pregnancy, participants noted the importance of trusting their obstetric (OB) provider and healthcare system to navigate the choices associated with prenatal genetic testing, and of relying on their healthcare provider for information and decision support. Many reported feeling confident in their provider because of a strong relationship and the trust that their provider would prioritise their needs. “*I would probably just go off of what my doctor said. I trust medical research, and I trust all my doctors. So, if I didn't know, I would just trust whatever the doctor is recommending me to do and what's in my best interest based on their knowledge*” (G1‐19).

Several participants expressed that their reliance on their OB provider for information was founded in the belief that they would provide accurate information needed to navigate testing their options in a way that aligned with their values and needs. *“I probably just rely on the doctor. Honestly, if it's too complicated for the normal person to understand, I probably would just trust the doctor's recommendation”* (G1‐22). These accounts suggest that trust may play a different role across varying levels of decisional complexity. Some participants prioritised their providers' recommendations over other sources, particularly given concerns about the inaccuracy and potential harms of Internet and AI‐based information found on PS&D. Participants cited concerns not only about the quality and potential for misinformation on websites and social media, but also *“anxiety”* that could arise from *“going down the rabbit hole”* on the Internet. *“I think the more knowledge you have, the correct knowledge you have, it's comforting. I would rather have my OB (sic) give me that information and give me that education than me going on the Internet and trying to figure out what all this means and getting the wrong information. That in itself is anxiety‐producing, you know?”* (G3‐20).

Participants noted that reliance on their OB provider increased as prenatal care decisions became more complex. This decision‐making point represented not just an increase in the volume of information they had to learn about and the need to make decisions in accordance with their values, but also the time‐sensitive nature of the decisions in which their ability to obtain reliable information from other sources was limited. The importance of this relationship was noted in the preference to have an abnormal result disclosed by their OB, even if they were not the content expert on the genetic condition, and in favouring the OB's presence in multi‐team discussions about care management. *“I've put my trust in someone as a provider. […] If it's the same OB for multiple [pregnancies], I trust this person. I want this person in the room. I want to hear those results from somebody that I have a rapport with*” (G3‐06).

As part of inquiry about factors that would strengthen trust with the healthcare provider, participants were asked specifically whether AI‐based tools could help support decision‐making and build trust in the context of care. These responses therefore reflect a targeted line of inquiry rather than an emergent theme arising spontaneously in the interviews. Participants indicated that AI‐based tools, such as chatbots, could be helpful when used as an electronic extension of their trusted healthcare provider, particularly in circumstances in which their healthcare provider was not immediately accessible. As one participant explained about the benefits of continuity of communication with their provider, *“I use chatbots a lot for certain things. So, I think that that [chatbot for PS&D options] would be helpful as well, because sometimes you'll get a result and although you can message your doctor about the result if you get the result like I got a result for a test the other night at like 11:00. Wow, nobody was available to answer 11:00 on a Sunday. To be able to send a message to a chatbot would be helpful*” (G1‐16). At the same time, participants emphasised that the usefulness of such tools was contingent on clinical oversight. Specifically, they suggested that AI‐based resources would only promote trust if the information provided was created, reviewed, or vetted by their healthcare professional, reflecting concerns about accuracy, reliability, and bias. *One participant stated, “I think, as long as it was created by and vetted by medical personnel*” (G1‐15). In this sense, trust in the provider may be extendable into the AI context when the tool is understood to convey information that has been endorsed by the healthcare team.

### 3.2 Trust As a Critical Component of Informed Decision‐Making

Participants discussed trust alongside their ability to make informed decisions about PS&D. Many discussed the need to establish a relationship with a provider before they felt comfortable asking the questions needed to gather information about their options. *“I think it's a little bit harder to trust until you form that relationship and that comfort level with somebody”* (G1‐03). For this participant, prior negative experiences with healthcare providers led her to delay seeking specific types of information from the provider until the relationship was established. *“I was kind of concerned because I've always had some issues with doctors in the past, not listening. So, I was pretty concerned about it at first, but after the first couple visits, I was pretty comfortable with my doctor. So, I listened to what he had to say, and all of my questions went to him” (G3‐03)*.

Having placed trust in their primary OB and their reliance on them for information, participants also reported hopes that providers would be neutral when PS&D discussions touched on personal and sensitive topics, such as disability and termination. “*As a health care provider, I think you should be the impartial person in the room and speak to both sides of the aisle. As a patient, I can graciously take that and say, ‘Thank you. I'll make my decision.' But I can understand why some providers would lean one side or the other because either they've done their research or, you know, have personal beliefs. It's such a hard line to navigate, right?* (G3‐18).

Participants recognised that such conversations could become more difficult as changes occur outside the patient‐provider relationship. That recognition included the increased complexity of prenatal and reproductive healthcare decisions: “*I think what's hard is abortion is such a touchy subject, it's so emotional, and then it's unfortunately so political. It's hard to have a neutral healthcare conversation without feeling* […] *That's OK if a provider does not personally believe in abortion or wanting to help with that. Then, that's their own choice. So then how can they get them to the right person who could have those conversations and not have a bias*?” (G1‐14). However, participants recognised that some providers may not have the skills or interest in navigating decisions with neutrality or prioritising the patient's needs above their own. “*My doctor specifically, she's not judgmental. Now I'm not sure how every doctor is. I'm sure there's going to be a couple bad apples here and there who are judgmental*” (G1–22).

### 3.3 Erosion of Trust By Perceived Provider Bias, Opinions and Judgement

Participants discussed how a provider's beliefs, opinions, and biases can influence discussions about PS&D, potentially negatively affecting patients' ability to make informed prenatal care decisions. Participants reported that trust in a provider developed over time but could be threatened or undermined if a patient perceived bias or judgement coming from that provider. Without trust, participants reported reluctance to engage with their provider to learn about prenatal genetic testing, particularly when those discussions pertained to personal and potentially sensitive topics of what would be considered acceptable options if a serious foetal abnormality were identified. Participants spoke of having a keen sense of when their opinions about the utility of prenatal genetic testing, disability, and termination differed from those of their providers, and that a sense of alignment or misalignment with their providers' opinions could profoundly affect communication and the exchange of information needed to support informed decision‐making. *“I think the best advice would be to give them as much information as you can and to be neutral in the way you provide the information. I think sometimes, when I'm given information, I know how my doctor feels about it. So that sways how I want to respond in the moment or how I'm feeling about it”* (G1‐08). Some participants noted that a sense of misalignment may significantly hinder communication. *“I kind of don't care what you're saying to me because I can tell what side you're on, and you're not staying neutral. I think that's really important in a healthcare setting, especially when there's, you know, mothers and babies involved”* (G3–16).

Provider biases were experienced in different ways. One subtheme concerned providers' assumptions and beliefs about the type and level of detail patients would want regarding prenatal genetic testing. “*Even though their job as a doctor, I am sure, is to stay pretty neutral no matter what. But I guess she [her provider] read my situation. You know, I'm excited to be pregnant. Maybe she knows I don't get anxiety easily. I don't get stressed out about those things. I don't know. Maybe she just knew that I didn't need to talk about that as an option. Maybe she just used her judgement on me as a person, and that went into her decision into giving me information or not, or maybe personal information weighed in her decisions to give me information or not*” (G1–08).

These assumptions included providers making determinations about which information a patient may want without the patient's input. *“I think probably it's become very routine; it's something that comes up in every pregnancy. So, maybe they feel that they don't have to explain it as much or go into detail as much or, you know, in my case, with it being my fourth pregnancy, they may assume that I already know or have a preconceived opinion about whether I want the test or not”* (G3–10). There were also concerns about the timing of information access and about providers deciding when a patient would want to learn about their options without the patient's input. *“They said they would address it if there were [an abnormal result] that came back, so I didn't even need to explore that option*” (G3–18). Some participants described situations in which providers' beliefs could direct care with little regard for individual patients under their care. “*The first pregnancy, they offered that [diagnostic testing] to us, and we didn't get it done because the doctor that did our ultrasound said that it was unnecessary to test for it because of the nuchal fold or something. He said that he was not concerned with it. So, doing further genetic testing wouldn't be necessary, essentially. It would be a waste of resources is what he presented it as”* (G3–20).

Another form of provider bias raised by participants was the potential for patients to judge providers in response to questions or disclosed preferences. Participants reported that concern led them to be cautious about sharing information about their preferences or beliefs. “*I always lean on the side of the more information, and the more that I share with my provider, the better they can take care of me. I know that, with what we're talking about, I might lean a certain way because I might be fearful of the judgement piece*” (G3–12). Fear of negative judgement from their provider led to a reluctance to discuss personal topics and concern that their preferences and beliefs might affect how a provider would share information with them.

I have known [my midwife] personally and professionally. We have a relationship. I felt very comfortable telling her [about my opinions if a genetic condition were identified], whether she asked about them or not. I could see either side of it because, what if I didn't know her as well as I did and have been seeing her as long as I have? Would I feel comfortable saying that to somebody else? In the back of their mind, would they be thinking about that when they saw me, knowing my opinion and maybe thinking about me differently? I would worry about the judgement a little bit if I didn't know my provider as well as I knew mine personally.

(G3–12)

Participants noted that fear of judgement could also influence what information they chose to raise during appointments or even how they spoke about a topic. “*When you're in that situation of even considering that [a positive diagnosis], you need somebody who would be not‐judgmental and be open to have that conversation with you and not shut it down because I unfortunately have a feeling there are practitioners out there that are just like, ‘Nope,' you know?” (G1‐17)*. Participants were also concerned about the fear or experience of feeling judged by their provider, with the worry that differing opinions may diminish the quality of prenatal care. In these cases, they might seek other sources of information, even if they realise those sources may not be as reliable as what a trusted provider can tell them.

Participants acknowledged that these other sources may also be biased. Still, this bias seemed more acceptable when they did not have a trusted provider. *“For this pregnancy, I would say it's more difficult since we just moved to the area from [state redacted], so I didn't have an OB (sic) set up until I got pregnant, so I'm not as familiar with this doctor. I've only interacted with her in my prenatal appointments, so I just feel more comfortable getting the answers myself”* (G2‐05). Another participant recognised the variable quality of information that they can access outside of their provider but also the need to achieve the trust to seek it from them: *“I think there's so much misinformation and there's just so many opinions out there, and having that direct contact to medical professionals to just ask questions when maybe you're not so comfortable…you're not in a position where you're comfortable enough yet to come face to face with somebody”* (G3‐18).

### 3.4 Building and Maintaining Trust Across Different Models of Prenatal Care Delivery

Participants also described several barriers to establishing a strong relationship with a new or existing provider during a new pregnancy, to foster the level of trust needed for informed discussions about prenatal genetics. For many, these barriers were primarily related to the structure of prenatal care delivery. In this case, participants referred to prenatal care models that included a patient seeing multiple providers during pregnancy and having limited time or opportunity to build a level of trust to have such conversations, particularly in early prenatal care. *“It's a person who doesn't know you. It's a screening visit and the patient isn't opening up with that person to the extent that that they will open up and have conversations with their physician…in the context of this conversation is a huge disservice to patients…Just the lack of opportunity for vulnerable conversations” (G3‐04*).

Reluctance to share and seek information with a provider was also related to group practice care models, in which patients have little control over whom they see within the group. Participants reported feeling “rushed” and that they did not have enough time or opportunity to establish the vital relationship they needed. *“I personally felt like that at some of my appointments, not with my preferred primary doctor, but I've seen other doctors when there wasn't availability for her. So that's when I felt like I was rushed. I didn't feel comfortable enough to really ask questions because I felt like it was being rushed.”* (G3‐14). Another participant observed, *“I think it's very easy for a provider to skip over something or maybe not fully say something that might be important*” (G3‐11). Some participants expressed concern about receiving care from a provider in the group who might not share their opinions, even if they had established a trusting relationship with their primary provider:

I think people would be afraid it [their opinions about terminating a pregnancy with a serious foetal abnormality] would be documented and kept and held against them. I know there are just some people who just aren't trusting of people, and especially medical professionals. […] I feel like they would feel like they're prejudged when they walk into that room.

(G1–17)

These concerns led some participants to call for an approach to minimise potential bias or judgements that could arise when seeing multiple providers during the prenatal care episode. *“I think a lot of it is just so hard because it's delicate conversations and figuring out, if you have a patient who screens positive for something, does the team have a good approach, like a standardised approach for how they're going to help that patient, so everybody's on the same page? Not that you want to script people, but sometimes that's helpful because maybe that helps with a little bit of bias”* (G1‐14).

## 4 Discussion

The findings of this study suggest that trust may be a key factor shaping how pregnant patients navigate prenatal genetic testing decisions. Participants described trust as influencing how they sought information, interpreted recommendations, and engaged in decision‐making about PS&D. When exploring components of the decision‐making process, we identified trust as a significant factor in patients' ability to navigate their options, informed by medical evidence and aligned with their values and needs. We recognise that trust in healthcare operates at multiple levels, including trust in individual clinicians, trust in healthcare teams and institutions, and broader systemic trust in the healthcare system as a whole [24]. Participants in our study primarily discussed trust in terms of interpersonal trust within the patient–provider relationship, as this form of trust most directly shaped how they sought information and made prenatal genetic testing decisions. This trust shaped the nature and detail of information exchange between the patient and provider, key components in supporting medical decision‐making that align with patients' needs and goals. While factors such as patients' communication preferences and information needs also contributed to the decision‐making process, they could not be engaged without a sense of trust. These findings shed light on the complexity of this type of decision‐making and on the need for multifaceted interventions that support patients through the prenatal genetic testing process and address their unique needs.

Trust and the patient‐provider relationship are well established as the foundation of effective healthcare communication [25, 26]. High‐quality therapeutic relationships characterised by trust, respect, and psychological safety enable patients to ask questions, disclose concerns, and integrate medical information with their values and priorities [27, 28, 29, 30, 31, 32, 33, 34]. This study's findings capture the extent to which patients depend on their primary OB to foster a therapeutic relationship and obtain accurate, unbiased, and timely information about their PS&D options and the role of trust in that process. Primarily, they want to trust that their clinician will provide accurate, unbiased information tailored to their needs. The unique context of prenatal care decisions has been described in other studies that characterise the nature of antenatal care decisions and the many individuals who may be involved in the decision‐making process (e.g., primary OB provider, obstetric specialists, partners, or other family members) [34, 35, 36, 37]. Many studies focus on the importance of shared decision‐making as a mechanism to optimise obstetric outcomes and methods to support it [37, 38, 39, 40, 41]. The findings suggests that trust is not merely an interpersonal backdrop to informed choice, but a core component that may shape how informed decision making is possible across different stages and levels of decisional complexity. We propose that trust should be integrated into frameworks of prenatal healthcare decision‐making.

One interpretation of these findings is that trust may operate in different ways depending on the complexity and stakes of the decision. In earlier decisions, such as whether to initiate testing, trust appears to function primarily as a facilitator of the shared decision‐making process that can foster informed PS&D decisions. At this stage, patients often weigh risks and benefits using relatively established, static factors, including maternal age, prior reproductive history (e.g., a prior pregnancy with a foetal congenital abnormality), and known test characteristics (e.g., sensitivity, specificity, positive predictive value). In these contexts, patients' trust in their provider and in the information and decision support they provide supports patients' engagement with information and clinicians, a primary component of shared decision‐making. However, as decision‐making may be more straightforward during initial consideration of the risks, benefits, and limitations of testing, trust may not be a prerequisite, as patients may be able to navigate these decisions on their own, even if they would prefer to rely on their provider. By contrast, as decisions become more complex and the stakes for the pregnancy increase, particularly with advancing gestational age, trust may take on a more enabling role. Under these conditions, patients may rely more heavily on their clinicians not only for information but also for guidance in interpreting uncertainty, navigating emotionally and ethically difficult choices, and moving forward with decision‐making. In these situations, patients may increase their reliance on their trusted provider as a reliable source of truth to bridge the gap between what they can learn and personally contextualise within a limited timeframe, as gestational age advances and windows for PS&D testing and posttest decisions close.

We propose that trust can support autonomy by enabling patients to make decisions that are consistent with their values, even when they rely on their provider's expertise to navigate the medical aspects of the choice. Patients may use their own priorities, beliefs, and preferences to anchor decision‐making, while trusting their clinician to provide, interpret, and contextualise information in light of those values. For this reason, the findings suggest that trust is not just a relational feature of healthcare delivery, but a mechanism that can enable autonomous and shared decision making. In this framework, an autonomous decision does not require patients to master all relevant medical information independently; rather, it may include the deliberate choice to rely on a trusted clinician to help navigate complex decisions in a way that remains grounded in the patient's own values.

The findings shed light on a link between patients' perception of a provider's conscious or unconscious biases and a hesitancy to engage in conversations about PS&D fully. Participants recognised the uniquely high‐stakes nature of PS&D decisions and the need to make informed choices that align with their values to avoid decisional regret. At the same time, they described a tension between their desire to trust and rely on their healthcare providers when making complex decisions and their concern about encountering bias, judgement, or assumptions when discussing sensitive topics such as genetic conditions, quality of life, and pregnancy termination. Study findings indicate that breakdowns in trust can undermine informed, values‐concordant decision‐making when concerns about provider judgement or bias prompt patients to seek information elsewhere, even when they recognise that alternative sources may be less reliable. This hesitancy was especially pronounced when considering the type of discussion that might take place following a diagnosis of a serious foetal genetic condition, when decisions are time‐sensitive and emotionally charged. Our prior work shows that some patients prefer to receive comprehensive information about PS&D and post‐diagnosis options at the outset of prenatal care, often before trust has been firmly established [42]. Relevant to this study, challenges related to trust and concerns about bias were evident across different prenatal care delivery scenarios, including within new clinical relationships where values and preferences had not yet been explored, in longstanding relationships where providers may have made assumptions based on prior pregnancies, and in group prenatal care models where limited continuity or time constraints complicated sensitive discussions. The findings of the study add to the literature on the impact of bias and discrimination on persistent disparities in maternal and neonatal outcomes and the critical need for strategies to address these issues to improve outcomes [43, 44].

**Figure 2:**
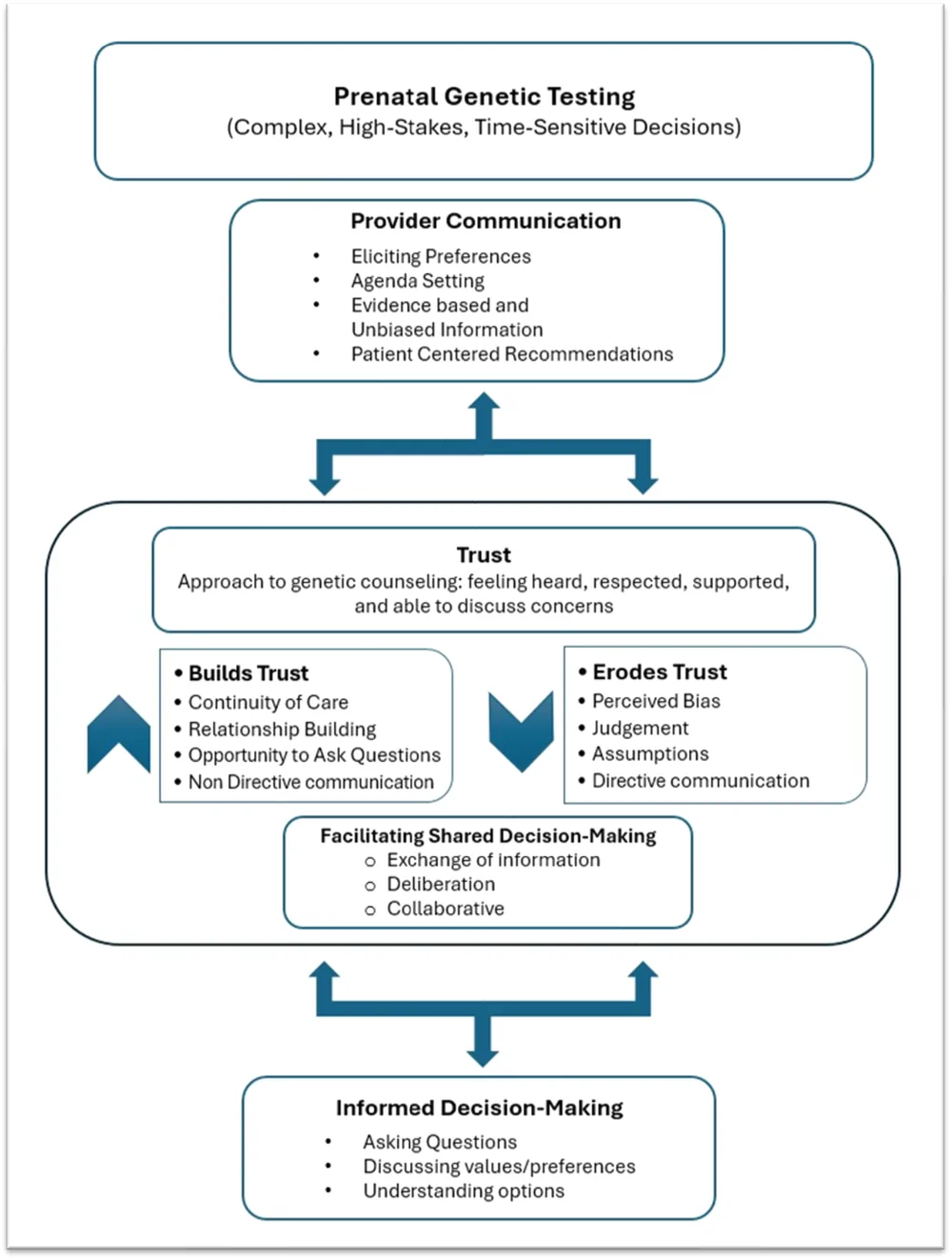
Conceptual relationship between trust and prenatal genetic testing decision‐making.

Together, the subthemes identified in this study suggest that trust is not a static characteristic of the patient‐provider relationship, but a dynamic process shaped by the interaction between two increasingly complex systems: (1) the complexity of prenatal genetic testing decisions and (2) the complexity of a changing approaches to prenatal care delivery. Participants described trust as a key mechanism linking provider communication and information exchange with informed prenatal genetic testing decisions. Trust was strengthened by continuity of care, relationship‐building, and perceived provider neutrality, while perceived bias, judgement and assumptions erode trust. Higher levels of trust facilitated question asking, discussion of personal values and preferences, and informed, values‐aligned decision‐making (Figure 2). As advances in prenatal genetics expand the number and complexity of available testing options, and as models of prenatal care continue to evolve, fostering trust becomes a critical intersection that enables meaningful communication, supports informed decision‐making, and helps ensure that decisions align with each pregnant patient's values, preferences, and needs.

Building trust is essential to establishing a therapeutic relationship that facilitates high‐quality communication and informed decision‐making. There is currently little guidance in the obstetric literature on the components of a trusting therapeutic relationship in prenatal care and on practical approaches to establishing and maintaining trust in this relationship during pregnancy. Thus, there is a need for mechanisms to build, communicate, and maintain trust across the prenatal period that are attuned to the highly preference‐based nature of PS&D decisions, in which the risk‐benefit ratio of engaging in or deferring testing shifts with gestational age. Such strategies must also address how to build and maintain trust across different modalities of prenatal care delivery, which may involve multiple providers from different backgrounds. This is a key concept, as participants described the potential for varying levels of trust and a sense of a trusting therapeutic relationship as they encountered different providers during their antenatal care.

The study's findings also suggest several approaches to prenatal care to set the stage for effective, patient‐centred informed decisions about PS&D. First, there is a need for additional research on factors that build and maintain trust in the prenatal patient‐provider relationship. Trust is a complex phenomenon that can be difficult to measure. It is time‐ and situation‐dependent, where trust may be specific to a particular aspect of care or result from a particular interaction with healthcare providers and may change over time or across situations. Trust dynamics may also vary across pregnant patient populations. For instance, pregnant patients who have experienced inequities, discrimination, and marginalisation when engaging in the healthcare system during or after pregnancy may have different notions of trust and how it may play a role in discussions with their healthcare provider and medical decisions [45]. Lack of trust in healthcare providers and systems may also translate into reluctance to participate in research examining aspects of the therapeutic relationship that may support or hinder access to patient‐centred, evidence‐based maternal care [46]. Thus, there is a need for instruments that measure the different factors that build and erode trust during prenatal care delivery. Approaches should evaluate aspects of various prenatal care delivery models and ensure continuity of patient‐centred care across providers and care settings. This includes mechanisms to recruit diverse patient populations into research, with specific steps and protections to support inclusion and help combat factors that erode or prevent the formation of trust. Second, there is an opportunity to improve healthcare communication to ensure patients' needs, preferences, and goals are prioritised at each stage of pregnancy and when engaging with different providers. One example is agenda‐setting at the start of prenatal care and at each visit, so patients' priorities are expressed and integrated into the visit [42, 47].

The need to overcome these challenges presents an opportunity to leverage technology to support healthcare communication while addressing the unique aspects of PS&D decision‐making. Artificial intelligence (AI) is one potential resource to support healthcare decisions [48, 49]. This idea also emerged when participants were specifically asked about decision aid resources at the conclusion of the interviews. For instance, tools using large language models (LLMs) could summarise patients' decision‐making needs and preferences during pregnancy, drawing on information from the prenatal care episode in the EHR. The provider could then use this information to help set the agenda at the start of the visit with the patient, collaboratively integrating the patient's decision‐making needs and preferences with the prenatal care milestones. Our prior research demonstrated the benefit of using a point‐of‐care tool to orient healthcare discussions at the start of prenatal care [50]. This intervention prompted patients to identify their decision‐making needs and preferences, then presented a summarised report of those responses to the provider to help structure the PS&D discussion during the visit, thereby reducing factors such as decisional regret [2]. An AI‐based tool could perform similarly, collating key events and patient‐centred information from the prenatal medical record and generating a summary that both providers and patients can use to set an agenda for the visit. This type of resource would require modifications to EHR and medical record templates to include discrete fields that capture and report patients' decision‐making preferences and other patient‐reported outcomes that are important for structuring effective healthcare discussions. This modification would enable better clinical care and future research to understand further how to strengthen the patient‐provider relationship. Technologies such as these present an opportunity for patients and providers to co‐create solutions that support patient‐centred prenatal care to improve obstetric outcomes. To be effective in this manner, AI‐based tools must address participants' concerns about the quality and reliability of information generated by digital technology observed in this study and how such input may affect prenatal care delivery. They must also address persistent ethical questions, privacy concerns, biases, and quality issues (e.g., misinformation and hallucinations) associated with their rapid growth [50].

While this study provides important insights into the PS&D decision‐making process, it also has limitations to consider. This was a qualitative study of one population of pregnant patients who self‐selected for the study. Thus, this population may reflect a subset of patients who had specific experiences or held different opinions than those who did not participate. Additional research is needed to elucidate factors such as trust, engagement, and interest in study participation. Most patients in this study were under 35 years of age, had a prior pregnancy, and identified as White and non‐Hispanic. While trends associated with maternal age and gravidity were not identified in this study, it will be important to examine how larger, more diverse populations across rural and urban settings describe their decision‐making needs and experiences. It is equally important to elucidate further how different patient populations may view and prioritise trust in providers and healthcare systems when navigating PS&D options and prenatal care decisions. It is well recognised that some maternal populations experience disproportionate rates of mistreatment during prenatal care delivery, with rates higher among women self‐reported as Black, Hispanic, or multiracial, and those without insurance. This is a factor that can profoundly affect trust, trust dynamics, and the role it may play in decision‐making. Given the continued observation of barriers to evidence‐based maternal healthcare, it is critical for future research to directly examine trust as a factor of current and past prenatal care experiences. In addition, this study was conducted in only one region in the United States, which may have needs and preferences reflective of local geographic, cultural, and socioeconomic/political trends. Future studies can address these limitations. Our population was asked to provide opinions on barriers to decision‐making, even if they had not personally experienced them. Our study focused specifically on trust within the patient–provider relationship; however, we recognise that this relational trust is embedded within a broader ecosystem of healthcare trust. Thus, additional research is needed to elucidate these areas and their interactions in the prenatal care setting.

This study suggests that trust may be a key component of informed decision‐making for prenatal genetic testing, helping patients interpret information, express their preferences, and engage meaningfully in complex discussions. Conversely, perceived bias, judgement, or fragmented care models undermined psychological safety and impaired patients' ability to obtain the information needed to make values‐concordant decisions. These findings highlight an urgent need for health systems to design or re‐evaluate current prenatal care delivery models to create modes that explicitly support trust formation across the care continuum. Strategies such as structured agenda‐setting, continuity‐enhancing workflows, communication training that centres on neutrality, and system‐level standards for discussing sensitive topics can mitigate the barriers identified by participants. Emerging technologies, including AI‐supported tools, may augment communication and decision support, but their integration must be approached carefully to avoid reproducing bias or eroding relational trust [51]. Ultimately, informed prenatal genetic decision‐making relies on trustworthy clinical relationships and trustworthy systems. Policies and innovations that prioritise trust, both interpersonal and structural, are essential for delivering patient‐centred prenatal care within an evolving genomic and reproductive landscape. This is particularly relevant to PS&D's rapid advances in genomics and the role of socio‐political factors in information delivery [52, 53]. The findings of this point to opportunities to support informed decision‐making through strategies and interventions that focus not only on patient and provider education but also on mechanisms to foster trust and improve healthcare communication across the current models of prenatal care delivery.

## Author Contributions

**Miller Finkelstein:** writing – original draft, writing – review and editing, formal analysis, data curation. **Christina Collart:** conceptualisation, investigation, writing – original draft, methodology, validation, writing – review and editing, formal analysis, project administration, data curation. **Lenicia Jenkins:** investigation, writing – original draft, writing – review and editing, methodology, validation, formal analysis, data curation. **Richard Frankel:** conceptualisation, investigation, funding acquisition, writing – original draft, writing – review and editing, methodology, validation, formal analysis, data curation. **Norah Crossnohare:** conceptualisation, investigation, funding acquisition, writing – original draft, writing – review and editing, validation, methodology, data curation, formal analysis. **John Bridges:** investigation, funding acquisition, writing – original draft, writing – review and editing, methodology, validation, formal analysis. **Monique Katsuki:** investigation, funding acquisition, writing – original draft, writing – review and editing, formal analysis. **Susannah Rose:** conceptualisation, investigation, funding acquisition, writing – original draft, writing – review and editing, methodology, validation, formal analysis. **Marsha Michie:** investigation, funding acquisition, writing – original draft, writing – review and editing. **Megan Allyse:** investigation, funding acquisition, writing – original draft, writing – review and editing. **Michael Rothberg:** investigation, writing – original draft, writing – review and editing, funding acquisition, methodology, formal analysis, validation. **Marissa Coleridge:** conceptualisation, investigation, funding acquisition, writing – review and editing, methodology, formal analysis. **Brownsyne Tucker‐Edmonds:** conceptualisation, investigation, funding acquisition, writing – original draft, methodology. **Ruth M. Farrell:** conceptualisation, investigation, funding acquisition, writing – original draft, writing – review and editing, visualisation, validation, methodology, software, formal analysis, project administration, data curation, supervision, resources.

## Ethics Statement

The study was approved by the Cleveland Clinic Institutional Review Board (IRB) and all components of the study were performed in accordance with the Declaration of Helsinki (as revised in 2013). All informed consent procedures were proposed and approved by the Cleveland Clinic IRB. Informed consent was obtained from all individuals included in this study.

## Conflicts of Interest

Dr. Rose is a consulting ethicist for Blue Cross Blue Shield Association (national nonprofit). The remaining authors state no conflicts of interest.

## Supporting information


Supporting File

## Data Availability

The data that support the findings of this study are available from the corresponding author upon reasonable request. Deidentified data that support the findings of this study are available from the corresponding author upon reasonable request.
